# Editorial Notes

**Published:** 1894-12

**Authors:** 


### Editorial Notes.

THE managing editor begs to say that the Index will appear in the January issue.

---

DRS. J. W. Griggs, of West Point, and Eugene Foster, of Augusta, favored the RECORD with a call last week.

---

DR. Samuel E. Milliken has been appointed general and orthopœdic surgeon to the Randall's Island Hospital, N. Y.

---

THE owners of the famous silver statue have written to the Cotton States and International Exposition management, asking the privilege of exhibiting the statue at the exposition.

---

DR. Louis McLane Tiffany, of Baltimore, was elected President of the Southern Surgical and Gynecological Association at the meeting held in Charleston. The Association will meet in Washington, D. C. next year.

---

DR. Thomas M. McIntosh, of Thomasville, has been appointed Principal Physician of the Penitentiary by Governor Atkinson. We congratulate our genial friend upon his appointment and wish him a successful administration of the office and extend him a cordial welcome to his new home.

---

THE sub-committee of the Committee on Finance of the Georgia Legislature's lower house has recommended, by unanimous vote, the resolution providing for an exhibit at the Cotton States and International Exposition. It is expected that most of the Cotton States will follow Georgia's example.

---

THE Cotton States and International Exposition Company has closed a contract with Chicago parties for the erection of a scenic railway. It will have an undulating track and cover 700 feet of space. The same concern had a scenic railway at the Midwinter Exposition in San Francisco, and it was one of the most popular features.

AT the Antwerp World's Fair Exposition, the Wm. R. Warner & Co., Chemists, of Philadelphia, Pa., were awarded the Grand Prize for the purity and excellence of their preparations.

---

WE take pleasure in congratulating our esteemed contemporary, the *Alabama Medical and Surgical Age*, upon the excellent production of a very fine photo-engraving of our friend, Dr. R. M. Cunningham, of Birmingham, the recently-elected President of the Tri-State Medical Association.

---

MESSRS. Sharp & Dohme have favored us with a handsome copy of their latest price-list. This catalogue gives a full description of the line of pharmaceutical preparations manufactured by this reliable house, and it would prove a most handy book of reference for our country friends who have to make out their orders for supplies from time to time. Address the above firm at Baltimore, New York, or Chicago.

---

THE following item appeared in the last number of the *Alienist and Neurologist:*

"ANTIKAMNIA.—The adoption of the monogram on the new tablets and the recall of all the old stock from the market, will prove of benefit to this firm and the many physicians who may hereafter desire to afford relief by its use. It will henceforth be sold only in tablet form."

This item appeared in all friendliness to Antikamnia, but we desire to call attention to the last paragraph as we have been advised by the proprietors of this excellent preparation that notwithstanding the change in the style of the package and form of the tablets, it in no sense changes Antikamia, nor withdraws "Antikamnia Powdered" from the market.

